# A Case Report of an Acute Severe Tachyarrhythmia Presentation With Underlying Cardiomyopathy in a Patient With Anabolic Androgenic Steroid and Thyroxine Misuse

**DOI:** 10.7759/cureus.62806

**Published:** 2024-06-21

**Authors:** Rabiu Momoh, Ahmed Hassan

**Affiliations:** 1 Critical Care, William Harvey Hospital, Ashford, GBR; 2 Anesthesia, William Harvey Hospital, Ashford, GBR

**Keywords:** anabolic androgenic steroid, substance abuse, body image perception, cardioversion, tachyarrhythmia, men’s health, public health, heart failure, thyroxine, cardiomyopathy

## Abstract

A description of an acute hospital presentation with severe tachyarrhythmia requiring multiple direct current cardioversions in a 45-year-old male bodybuilder with underlying cardiomyopathy possibly caused by long-term anabolic steroid abuse and more recent thyroxine misuse is described. A review of the literature regarding the above associations was also done. This case report further adds to the literature regarding the harmful effect of androgenic anabolic steroid misuse (with the added effect of thyroxine misuse in this case) on the heart.

## Introduction

There is increasing evidence in the literature of the significant debilitation and possible mortality that can result from anabolic steroid misuse, causing cardiac dysfunction or heart failure. Male individuals in their productive age groups are more affected by this problem. The summative or multiplier adverse effects on the heart from combining anabolic steroids with other abused substances (in this case, thyroxine) are also being increasingly described in the literature [1]. The National Athletic Trainers’ Association in the USA recognized the impact of anabolic androgenic steroids (AAS) abuse on the body’s multi-organ systems in their position paper that reviewed and summarized the best scholarly evidence regarding AAS abuse [2]. Gagnon et al. remarked that about three out of 100 persons worldwide in their lifetime would have abused AAS [3]. We hope to add to the body of literature regarding the above public and men’s health problems to curb the avoidable health complications described above.

We present the case of a middle-aged male with a long history of anabolic steroid misuse and a more recent history of thyroxine abuse who presented with severe tachyarrhythmias requiring multiple cardioversions and positive echocardiographic findings of significant left ventricular impairment.

## Case presentation

**Figure 1:**
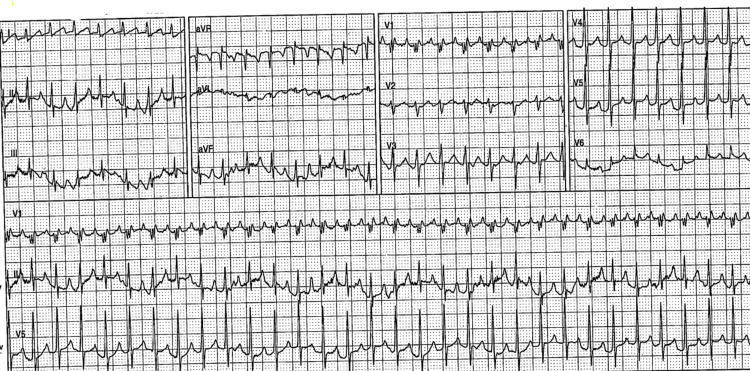
12-lead ECG showing SVT in patient SVT, supraventricular tachycardia

A 45-year-old male lorry driver, who was on a road journey from Spain to England, was brought in by ambulance crew to the hospital after he had complained of severe palpitation. He was noted to have severe tachycardia (a heart rate between 190 and 200 beats per minute) on evaluation by the ambulance crew, with a supraventricular tachycardia (SVT) rhythm noted on the 12-lead ECG done (Figure 1). There was no resolution of the SVT with the Valsalva maneuver. On arrival at the hospital, his heart rate was 190 beats per minute and his blood pressure was 112/50 mmHg, but he was peripherally cold. Initial fluid boluses up to a total of 1.5 liters were done without reversal of his SVT. Three IV adenosine doses (6 mg, 12 mg, and 18 mg) given in the emergency department had no effect on the patient. He was sedated, and direct current cardioversion (DCCV) was done with the return of his heart rhythm to sinus rhythm.

Post-cardioversion, he was noted to be tachypneic, desaturating, in acute delirium, and combative. His oxygen requirement ramped up to nearly 100% FiO2 to maintain pulse oximetry saturation around 90-92%. The chest X-ray showed bilateral diffuse opacities with bilateral infiltrates. An urgent CT pulmonary angiogram revealed no evidence of pulmonary embolism and revealed bilateral dependent consolidations, bilateral ground glass changes, evidence of a full stomach, bilateral pleural effusions, septal thickening, and reflux of contrast into the inferior vena cava (IVC) in keeping with fluid overload. A quick bedside echocardiography revealed severely impaired left ventricular function (video is unavailable). A rapid-sequence endotracheal intubation was done.

The patient had further episodes of atrial fibrillation (AF) with rapid ventricular response, compromising blood pressure readings in the ICU, and five further DCCVs were done. IV amiodarone 300 mg was loaded, and a maintenance IV infusion of 900 mg was run over a 24-hour period, and the infusion was repeated over another 24-hour period. The patient was then stepped down to a weaning regime of amiodarone delivered enterally.

On a collateral review of the patient’s past medical history, he was noted to have dilated cardiomyopathy that spanned back to when he was 22 years old, attributed to his use of anabolic steroids while training to be a professional bodybuilder. The patient had persisted with the use of testosterone injections (totaling four units per week) until the current presentation, despite being on a heart failure treatment regime (sacubitril/valsartan combination, bisoprolol, and dapagliflozin). The patient was also noted to be misusing non-recommended levothyroxine (dose unknown) in addition to the anabolic steroid within two years of the current hospital presentation. He was also noted to have had a history of paroxysmal AFs and had an AF ablation procedure done seven years prior to this current presentation. He reported having been considered for a cardiac device before his trip out of Spain. Further collateral history-taking revealed that the patient had had an ICU admission back in Spain about 30 months prior with almost the same presentation with severe SVT accompanied by cardiogenic shock and acute renal and hepatic failures. He had no smoking or alcohol drinking history.

On examination in the ICU following the episodes of cardioversion, he was sedated and mechanically ventilated. He had bilateral inspiratory and expiratory crackles up to both upper lung zones. He did not exhibit peripheral edema. However, he required a high FiO2 of 80% to achieve the target oxygen saturation of 92% or higher. His heart rate was 100 bpm, and his BP was 115/62 (79), which was unsupported. His abdomen was soft and non-tender.

**Figure 2:**
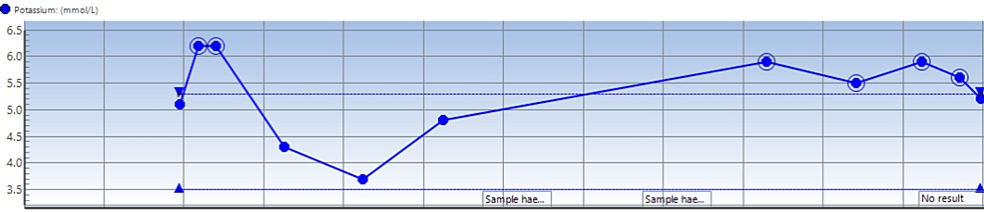
Serum potassium trend through the patient’s admission Each vertical line from the left-hand side to the right represents progressive days into the patient’s admission.

**Figure 3:**
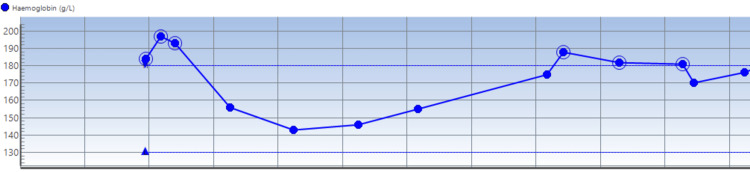
Serial hemoglobin trend through the patient’s hospital admission Each vertical line from the left-hand side to the right represents progressive days into the patient’s admission.

**Figure 4:**
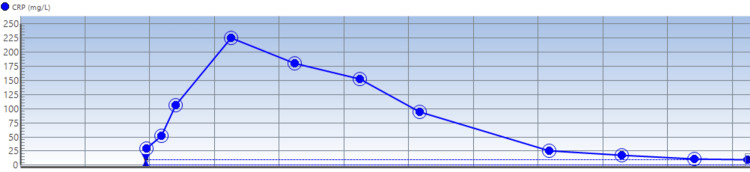
Serum CRP trend through the patient’s admission Each vertical line from the left-hand side to the right represents progressive days into the patient’s admission.

Notable findings on blood studies done at admission were the presence of hyperkalemia of 6.2 mmol/l (Figure 2), which was initially refractory to medical correction, and then the patient was dialyzed over a 24-hour period in the ICU. He was noted to have a raised hemoglobin level of 180 mg/l (ref: males: 138-174 g/l) (Figure 3). The serum troponin check was 88 ng/l, CRP 29 mg/l (Figure 4), WCC 18.8 × 10^9^/l, ALT 150 U/l (other liver function tests were normal), serum creatinine 140 umol/l, and eGFR 52 mL/min/1.73 m^2^. His thyroid function test was a normal study. A blood culture study yielded no growth after 48 hours of incubation. The serum procalcitonin level was 0.25 ng/ml (ref: for active infection >0.25 ng/ml) at admission. HBA1C was 29 mmol/mol (ref: <40 mmol/mol).

**Figure 5:**
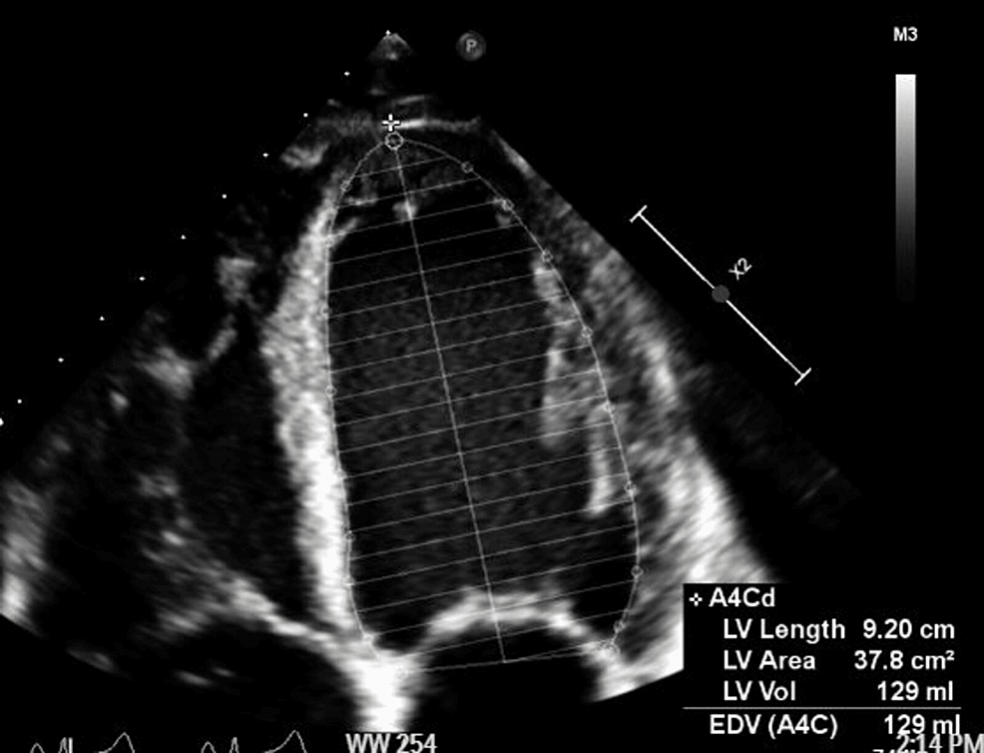
Apical four-chamber view in diastole revealing left ventricular measurements

**Figure 6:**
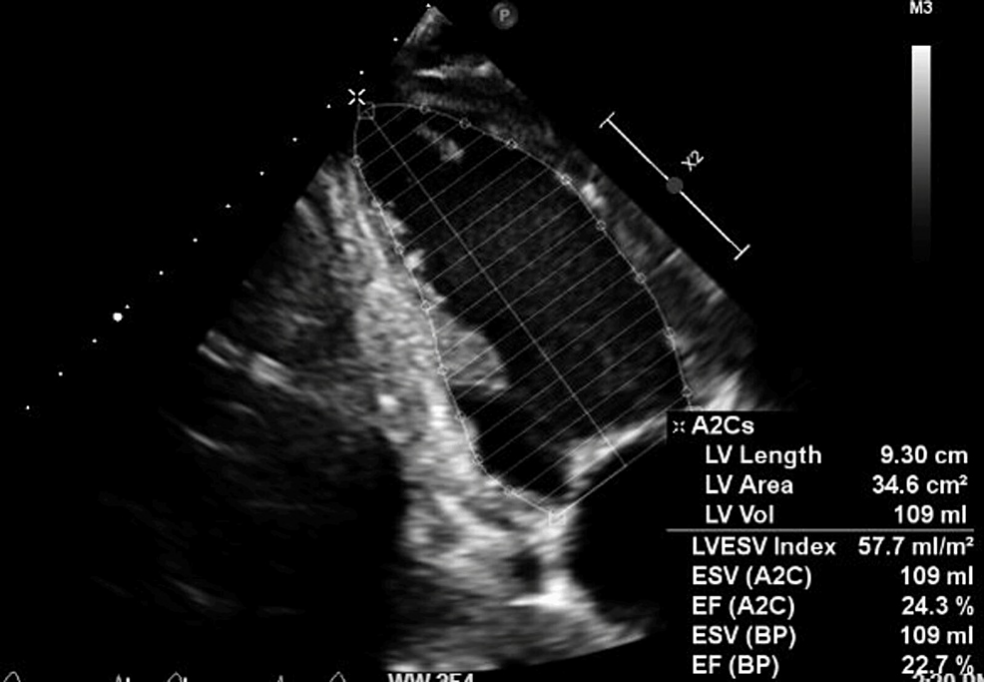
Apical two-chamber view revealing left ventricular measurements in systole and a reduced ejection fraction measurement

**Figure 7:**
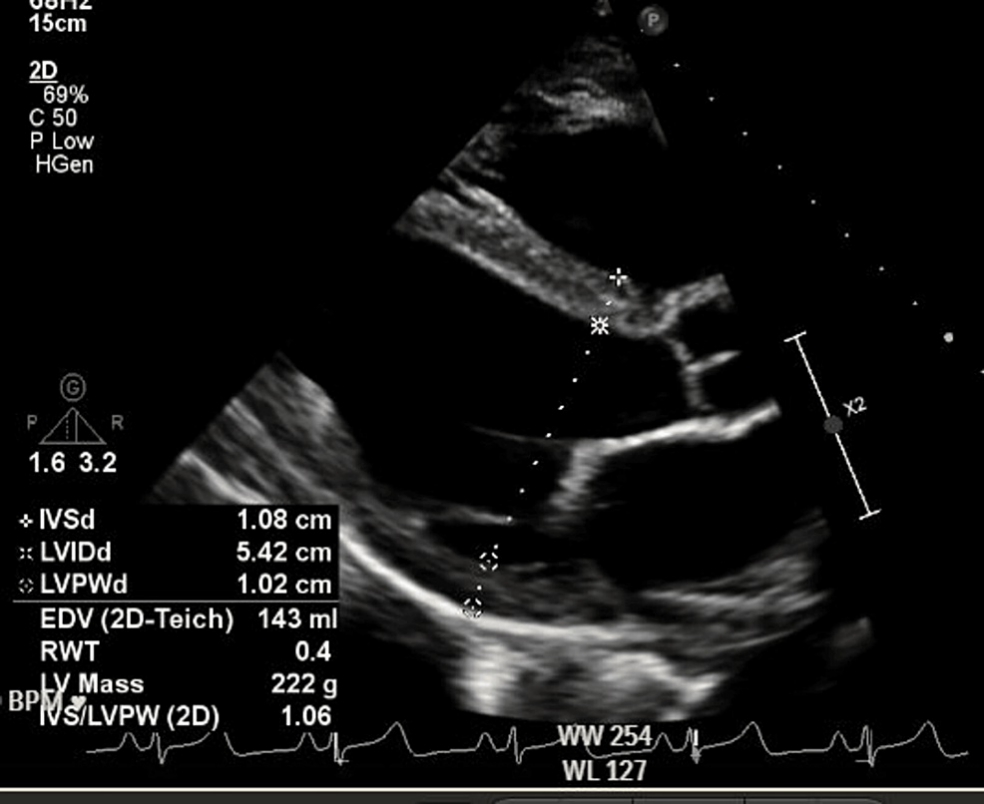
Further cardiac measurements in the parasternal long-axis view

A subsequent formal echocardiography (Figure 5, Figure 6, Figure 7, Video 1, Video 2, Video 3, Video 4, Video 5, Video 6, Video 7) revealed a mildly dilated left ventricle (LV) with severely impaired systolic function. The estimated visual LV ejection fraction was 25% ± 5%. Additionally, he had a stroke volume index of 20 ml/m^2^. He had a globally hypokinetic LV wall with impaired LV longitudinal function. He had an overall normal-sized RV with impaired RV function. He had a dilated left atrium and a borderline-dilated right atrium. He had a mild to almost moderate mitral regurgitation and a mild tricuspid regurgitation. A restrictive mitral valve inflow pattern, suggestive of elevated LV filling pressures, was noted. The aortic valve was tricuspid, and its cusps were thin and mobile, with good excursion. No aortic stenosis or aortic regurgitation were noted. The mitral valve leaflets and chordae were mildly thickened with good excursion. There was no mitral valve stenosis. The tricuspid valve leaflets were trivially thickened with good excursion. There was no tricuspid stenosis but mild tricuspid regurgitation. Pulmonary artery pressure measured by acceleration time was 26.7 mmHg (normal: <25 mmHg). The pulmonary valve leaflets were thin and mobile, with good leaflet excursions. There was no pulmonic valvular stenosis. Trivial pulmonary regurgitation was seen. The aortic root was of normal size. Dilated IVC was noted but unable to demonstrate collapse. There was a trace of pericardial effusion on the echocardiogram.

**Video 1:** Apical four-chamber view revealing significantly impaired LV contractility and dilated left atrium LV, left ventricle

**Video 2:** Parasternal long-axis view

**Video 3:** Parasternal short-axis view

**Video 4:** Subcostal view

**Video 5:** View of the IVC, distended, non-collapsing IVC, inferior vena cava

**Video 6:** Upper mild mitral valve regurgitation demonstrated

**Video 7:** Mild tricuspid valve regurgitation demonstrated

The patient was treated with IV amoxicillin and metronidazole in a bid to treat aspiration pneumonitis. A blood culture study yielded no growth after 48 hours of incubation, but non-direct lavage of the lower airways yielded *Klebsiella pneumoniae*,* Candida albicans*, and normal respiratory flora. Streptococcal pneumonia, COVID-19, and flu screens were negative. Screening for *Legionella *and *Mycoplasma *was negative as well. The patient’s oxygen requirement was met, and he was successfully extubated onto a high-flow nasal cannula of oxygen after 48 hours in the ICU. His regular bisoprolol and dapagliflozin were restarted on Day 3 in the ICU, and the sacubitril/valsartan combination was added on Day 4. He was stepped down to a cardiology ward bed on Day 7. He received further monitoring and optimization on the cardiology ward in preparation for his repatriation back to Spain. His use of the sacubitril/valsartan combination was discontinued because of a persistently mildly elevated serum potassium value (5.5-5.9 mmol/l). At the time of his discharge, his medication list included PO amiodarone 200 mg OD, PO bisoprolol 2.5 mg OD, and PO dapagliflozin 10 mg OD.

The patient’s education regarding his condition was facilitated. He was informed that he would need to stop testosterone and thyroxine misuse, as they were likely the cause of his cardiomyopathy and severe tachyarrhythmia presentations, and was told to inform the necessary driving authorities regarding his heart failure, who would need to assess his ability to drive (including driving heavy goods vehicles). His case was discussed with the managing cardiologist in Spain, and his repatriation was facilitated on Day 10.

## Discussion

There is an increasingly wide reporting of AAS abuse problems across a wide range of countries in the literature. This abuse problem occurs in a bid by individuals to enhance their physique, build stronger bodies, and enhance performance, and some abuse these anabolics for leisure. AAS are noted to be abused alone or in combination with other abused substances. Part of the untoward consequences of this abuse problem is the toxic effect on the heart and the ensuing heart failure, dysrhythmia, or sudden cardiac death that can result [1]. The index case under review was an AAS and thyroxine misuse situation with attendant structural and functional cardiac dysfunction.

Different cardiac pathologies have been reviewed in relation to AAS abuse. Hypertrophic and dilated cardiomyopathy have been described in relation to it [1]. Placci et al. described an acute case of Takotsubo cardiomyopathy in a gentleman in his mid-20s who abused nandrolone and stanozolol. They noted a resolution of his left ventricular dysfunction within a week following hospital treatment and cessation of AAS misuse [4]. Severe tachyarrhythmias have also been described in relation to AAS abuse. Adhikari et al. published the case of a gentleman (54 years old) with a history of substance abuse who presented with AF and attendant impaired left ventricular systolic impairment and went on to have an ablative procedure to treat the AF. They noted an improvement in the patient’s left ventricular ejection fraction following further guideline-directed medical management [5]. In certain cases, life-threatening arrhythmias and sudden cardiac death have been described. The proposed mechanisms of the cause of sudden cardiac death in AAS abusers include the vasospasm theory, the thrombogenic theory, the atherosclerosis theory, and possibly direct myotoxicity [6]. Barbosa Neto et al. concluded that AAS abuse is associated with sympathetic modulation and vagal attenuation following their study assessing for structural and autonomic dysfunction among three groups of a total of 45 subjects (group 1: bodybuilders who abuse AAS (n = 15), group 2: bodybuilders who do not use AAS (n = 15), and group 4: sedentary control subjects (n = 15)). They also noted that cardiac measurements, such as the interventricular septal thickness, LV posterior wall thickness, and relative diastolic wall thickness, were higher in the group of bodybuilders who abused AAS than in bodybuilders who did not abuse AAS and in sedentary controls (p < 0.001) [7].

The reporting of the reversibility of the cardiac pathology resulting from AAS abuse varies in the literature. Gul and Shahid reported the reversal of AAS-induced heart failure features after nine months in a 47-year-old female who had leisurely abused AAS for a couple of months prior to the initial hospital presentation for heart failure [8]. Angelini et al. described unreversed cardiomyopathy after six months despite treatment and cessation of further AAS abuse in a gentleman in his early 30s. The patient was noted to have abused AAS and growth hormones and had a hard gym routine prior to his initial hospital presentation for heart failure [9].

The index patient under review had misused AAS (testosterone) over an estimated period of two and a half decades and had a more recent introduction of thyroxine to this. The evident finding of significantly impaired left ventricular function on echocardiography is presented. A mild-to-almost moderate mitral regurgitation with a dilated left atrium was noted on echocardiography. His presenting complaint was severe palpitation, and he was assessed as having severe tachyarrhythmic rhythms (SVT and later fast AFs) that required electrical cardioversion. There was persistent AAS misuse in the patient despite knowledge of heart failure and being on treatment. Serial echocardiogram studies done throughout the course of the patient’s hospital stay could have demonstrated the impact of the ensuing pneumonitis that the patient developed on his echocardiographic parameters and clinically on his heart failure.

An unknown amount of thyroxine (that was not medically recommended or indicated) was consumed daily by the patient as an adjunct to testosterone (four units/week) in a bid to enhance his body image and physical performance. He had a normal thyroid function test done on the blood. Thyroid-induced cardiac dysfunction could be a possibility in this patient, but the patient had a normal thyroid function test on assessment and had a pre-dating history of cardiac impairment prior to the onset of the use of thyroxine. Thyroxine could, however, be a cause of agitation and tachyarrhythmia (including AF). Thyroxine and growth hormone, alongside AAS, were the abused substances quoted in a questionnaire survey of 375 gym users done between November 2012 and February 2013 in Amman, Jordan [10].

Sagoe et al., in a systematic review publication of 50 publications emanating from 10 countries, highlighted the tendency for AAS abusers to engage in polypharmacy with licit and illicit drugs such as growth hormone, cocaine, thyroxine, insulin, human chorionic gonadotropin, amphetamine/meth, clenbuterol, and ephedra/ephedrine. Analgesics/opioids, nutritional supplements, and diuretics are some other classes of co-abused substances with AAS. These co-substances with AAS are either used to potentiate the effects of AAS, lower the side effects of AAS, for recreational purposes, or for sexual enhancement [11].

The diagnosis of AAS-induced cardiomyopathy should be a diagnosis of exclusion, which can be corroborated with a clinical history finding of positive anabolic steroid use. A physical examination may reveal features of heart failure. Blood studies, electrocardiograms, and radiological investigations (chest X-ray, transthoracic echocardiography, and cardiac CT/MRI) will be useful in the assessment of AAS-induced cardiomyopathy. Coronary angiography studies may be needed to exclude ischemic causes of cardiac dysfunction in these patients [1]. From the history of the patient, his family, and his doctor in Spain, there was a shared knowledge that the patient’s heart failure diagnosis and treatment predated his trip to England and were attributed to his longstanding use of AAS. Further investigations such as cardiac MRI, coronary angiography, and possibly genetic studies would have been done if the patient had more time under the UK health system.

Guideline-directed treatment for heart failure may be needed in the treatment of AAS-induced cardiomyopathy. Cardiac devices such as pacemakers, implantable cardioverter defibrillators, or both may be needed in patients with severe or life-threatening arrhythmias. Left or right ventricular assist devices could be considered in certain patients as a bridge to receiving a heart transplant or while undertaking a watchful wait for a self-recovery of the heart’s function with medical treatment. Heart failure programs would ideally want patients to quit further substance abuse to be considered eligible for heart transplants [1].

## Conclusions

A rare description of an acute hospital presentation with severe tachyarrhythmia in a patient who misused AAS and thyroxine despite a prior assessment of AAS-induced heart failure has been made. AAS abuse or misuse still remains an avoidable cause of heart failure. Educating the public and community stakeholders about this problem needs to be done.
